# Microtubule Dynamics and Cancer

**DOI:** 10.3390/cancers14184368

**Published:** 2022-09-08

**Authors:** Andreas Ritter, Nina-Naomi Kreis

**Affiliations:** Obstetrics and Prenatal Medicine, Department of Gynecology and Obstetrics, University Hospital Frankfurt, J. W. Goethe-University Frankfurt, Theodor-Stern-Kai 7, D-60590 Frankfurt, Germany

Microtubules (MTs) are highly dynamic key components of the cytoskeleton composed of alpha- and beta-tubulin heterodimers. Microtubule dynamics are tightly regulated by the “tubulin code”, various microtubule-associated proteins, kinases, and phosphatases. Proper regulation of MT dynamics is not only important for mitosis and faithful chromosome segregation but also for cell signaling, trafficking, cell migration, and ciliogenesis. Defects in spindle assembly or the separation of the duplicated chromosomes into daughter cells may lead to cell death or genomic instability, causes for diseases such as developmental disorders and cancer. Given their indispensable role in cell division and in diverse cellular processes, MTs have served for decades as pharmacologically validated and attractive targets for cancer therapy. Understanding how the MT cytoskeleton is formed and regulated in somatic and malignant cells will help us to improve treatment strategies for cancer patients. For this purpose, the identification of proteins that modulate the MT network could lead to a better understanding of chromosome instability and tumor progression, and provide additional prognostic information for the selection of adequate anti-cancer therapies for patients to significantly optimize clinical outcomes. Therefore, this Special Issue focuses on how microtubules and their MT-associated proteins (MAPs) regulate MT nucleation, migration, chromosome congression, apoptosis, and autophagy in various cancer entities as well as how these structures can be used as future therapeutic targets.

The Special Issue “Microtubule Dynamics and Cancer” of the journal Cancers attempts to elucidate the regulatory mechanisms and functional consequences of MT dynamics in carcinogenesis and to find novel therapy approaches. In cells, MTs nucleate from centrosomes, called microtubule organizing centers (MTOCs) and γ-tubulin, which forms multiprotein complexes, is the essential hub for MT nucleation [1]. The γ-tubulin ring complex (γ-TuRC) is an efficient MT nucleator that requires additional centrosomal proteins for its activation and function. Centrosome aberrations are hallmarks of human cancers and evidence suggests that there is a dysfunction of centrosomal MT nucleation in cancer cells, whereas the mechanisms of MT nucleation in normal and cancer cells remains elusive. The review article by Dráber and Dráberová highlights the recent work on the high-resolution structure of γ-TuRC and MT nucleation. They discuss the effects of γ-TuRC protein dysregulation on cancer cell behavior and describe new compounds targeting γ-tubulin. Drugs that inhibit γ-TuRC functions or non-essential γ-TuRC proteins may provide an alternative to MT targeting agents in cancer chemotherapy [1]. In addition, the MT cytoskeleton is crucial for cell motility and migration by regulating multiple cellular activities such as transport and endocytosis of key components of focal adhesions (FA) [2]. The interesting work from Moon et al. utilizes a CRISPR/dCas9 system to address the functional role of the important MT depolymerase mitotic centromere-associated kinesin (MCAK/KIF2C) in regulating cell migration and invasion by modulating MT dynamics and FA turnover. Both up- or downregulation of MCAK led to reduced cell motility and poor migration in malignant as well as benign cells. Specifically, MCAK’s deregulation impaired the FA protein composition and phosphorylation status of key FA elements including focal adhesion kinase and paxillin, interfered with a proper spindle and chromosome segregation, disturbed the assembly and disassembly rate of FAs, delayed cell adhesion, and compromised the plus-tip dynamics of MTs, displaying the crucial role of tightly regulated MT dynamics in mitosis as well as interphase [2]. Additionally, the Special Issue contains further an exciting review article concerning the Casein Kinase 1 (CK1) family and its function during tumor progression, the cell cycle, MT modulation and transport [3]. Several CK1 isoforms localize to the centrosome and MT asters, which implicates regulatory functions in MT dynamic processes. Being localized to the spindle apparatus during mitosis, CK1 directly modulates MT dynamics by phosphorylating tubulin isoforms. Since CK1 plays a vital role in chromosome segregation, further analysis revealed that the RBP-J-interacting and tubulin-associated (RITA1) protein contains multiple putative CK1 phosphorylation sites. Modulating CK1 activity could be an interesting therapeutic approach for a multidrug treatment against tumor development. The therapeutic role of CK1-specific inhibitors is already investigated in preclinical studies for pancreatic cancer, colorectal cancer, breast cancer, skin cancer and leukemia, and phase I clinical trials for leukemia [3]. In addition to these MAP and kinases specific topics, Vona et al. describes the general function of MT-based mitochondrial dynamics in interphase cells as a valuable therapeutic target in cancer [4]. Mitochondria play a key role in several fundamental cellular functions that may be impaired in cancer cells. Mitochondrial dynamics are driven by MTs and the MT-associated motor proteins dynein and kinesin. They describe MT-dependent mitochondrial dynamics including MT fission and fusion, mitophagy, intracellular mitochondrial trafficking and the role of MTs in mitochondria transfer. Interestingly, exchange of mitochondria between stromal and cancer cells via tunneling nanotubes, for example, leads to cell survival, increased proliferation, and drug resistance. They also discuss the role of mitochondrial dynamics in cancer therapy. Therefore, understanding the molecular mechanisms that regulate mitochondrial trafficking can be important for identifying new molecular targets in cancer therapy [4]. Another study focuses on MT-based cellular structures called “microtentacles” (McTNs) that may influence the metastatic potential and chemoresistance profile of free-floating cells [5], first described in detached breast cancer cells. The development of chemoresistance to paclitaxel and carboplatin represents a major therapeutic challenge in ovarian cancer, a disease frequently characterized by malignant ascites and extrapelvic metastasis. Reader et al. investigated whether ovarian cancers exhibit McTNs and characterized McTN biology. Among all tested ovarian cancer cell lines, up to 30% of cells expressed McTNs. The metastatic potential and MT-stabilizing post-translational modifications correlated with the length and number of McTNs. McTNs are likely involved in key aspects of ovarian cancer metastasis. The authors therefore suggest that these structures may represent a new therapeutic target for ovarian cancer [5]. Another promising therapeutic approach is described by Rajendraprasad et al., who analyzed the effects of the mut-T homolog-1 (MTH1) inhibitor TH588 that inhibits tubulin polymerization in vitro and interferes with MT dynamics in interphase and mitotic cells, compared to low-dose nocodazole [6]. Both drugs stabilized MTs within the mitotic spindle, leading to premature formation of kinetochore-MT end-on attachments on uncongressed chromosomes. This caused mitotic arrest, ultimately resulting in cell death or cell division with congression defects. Both of these cell fates could contribute to the selective effect associated with the activity of TH588 in cancer cells, besides its inhibitory effect on MTH1. Strikingly, these reported effects appear to be specific for the tumor cell lines HeLa and U2OS, whereas the non-tumor cell line RPE-1 showed only a minor cell cycle arrest upon treatment with TH588 [6].

Another focus of this Special Issue are MT post-translational modifications (PTMs) that may influence the success of cancer therapy. The work of Trisciuoglio et al. discusses the crucial role of the “tubulin code”, generated by the choice of different α- and β- tubulin isoforms and tubulin post-translational modifications, in determining multitude cellular processes and different cancer phenotypes including metastatic cell migration, drug resistance, and tumor vascularization, and the influence of modulating tubulin-modifying enzymes on cancer cell survival and aggressiveness [7]. They further discuss the role of MT dynamics in autophagy, especially the role of different tubulin isoforms and PTMs in the autophagic process. Modulation of the activity of tubulin-modifying enzymes has clearly been shown to impact autophagy and the lysosomal-mediated degradation pathway. However, the stimulation of autophagy by interfering with PTMs increased survival rates in cancer cells, whereas inhibition of the autophagy flux induced apoptosis, reflecting the urgent need for further research to pave the way for novel cancer treatment options [7], as reasoned by the authors. In support, the research article by Mathias et al. describes the role of the tubulin carboxypeptidase (TCP) as an important tubulin detyrosinase, which is of particular interest in breast epithelial and breast cancer cells [8]. This study reveals that deTyr-Tub is tightly regulated in normal breast epithelial cells, and that excess deTyr-Tub promotes apoptosis. The immortalized breast epithelial cell line MCF10A underwent apoptosis following transfection with TCP constructs, but the addition of oncogenic KRas or Bcl-2/Bcl-xL overexpression prevented subsequent apoptotic induction in MCF10A. Whereas the TCP transfection in invasive breast cancer cell lines, MDA-MB-231 and Hs578t, led to increased levels of detyrosinated tubulin, enhanced focal gelatin degradation and resulted in enhanced tumor cell invasion, further strengthen the crucial role of PTMs in cancer progression [8].

The conclusive review article by Wordeman and Vicente engage microtubule-targeting agents (MTAs), one of the most successful first-line therapies in cancer treatment [9]. They illustrate how these agents bind to MTs, they summarize the molecular mechanisms behind MTAs function, and they describe their impact on neurodegenerative diseases and cancer, and the promising new therapeutic applications of these classic drugs. Thereby, they explain the function of MTs in cilia assembly, cell division, axonal transport, cell motility and polarity, as well as adhesion. As MTs are connected to these vital cellular functions, MTAs are linked to well-documented toxic effects against normal tissue. Nevertheless, more research into how dynamic MTs impact broad cellular activities may enable greater effective use of MTAs against human diseases [9]. Laisne and colleagues present such an important study [10]. They recently developed a cell-based assay that quantifies the cellular MT content. In their study they analyzed the effect of four well-characterized microtubule depolymerizing agents (MDAs) on the kinetics of tubulin assembly in vitro, on the cellular MT content and on cell viability, bridging the gap between in vitro tubulin assays and cell viability assays. Their newly designed technique might help to find novel potent MT poisons, which are suited for cancer therapy [10]. As example, epithelial ovarian cancer (EOC) is a cancer type, which would greatly benefit from novel therapeutic options, since this type is often associated with a resistance toward taxanes and epothilones, described by Tymon-Rosario et al. in their review article [11]. The first-line therapy option for woman with EOC consists since multiple decades of a combinatory approach including cytoreductive surgery as well as a platinum and taxane-based chemotherapy. However, EOCs develop often a resistance to platinum- and taxane-based drugs. Therefore, epothilones (ixabepilone) gained attention as an alternative treatment option, because they may retain activity in these resistant tumors due to a highly potent MT stabilization. This hypothesis of a beneficial use of ixabepilone was supported by recent clinical data of platinum-resistant or refractory ovarian cancer. Their results strengthen the role of MT-interfering drugs as mono or combinatory treatment option in EOCs. Furthermore, several ongoing clinical trials will help to investigate the potential of new MTAs and hopefully to establish these drugs as a standard therapy option for EOC. This reflects the utmost importance to investigate the cellular processes connected to MTs, apoptosis and the precise molecular signaling of MTAs to improve the clinical outcome of EOC patients [11]. 

We hope that the readership enjoys this Special Issue of Cancers, that they become informed about the role of MTs, MT dynamics, MAPs, MT-interfering drugs, and their roles in cancer therapy. More work is needed to advance the field with regards to rationally combining therapies targeting MT dynamics with other agents.

## References

[1] Draber P., Draberova E. (2021). Dysregulation of Microtubule Nucleating Proteins in Cancer Cells. Cancers.

[2] Moon H.H., Kreis N.N., Friemel A., Roth S., Schulte D., Solbach C., Louwen F., Yuan J., Ritter A. (2021). Mitotic Centromere-Associated Kinesin (MCAK/KIF2C) Regulates Cell Migration and Invasion by Modulating Microtubule Dynamics and Focal Adhesion Turnover. Cancers.

[3] Roth A., Gihring A., Bischof J., Pan L., Oswald F., Knippschild U. (2022). CK1 Is a Druggable Regulator of Microtubule Dynamics and Microtubule-Associated Processes. Cancers.

[4] Vona R., Mileo A.M., Matarrese P. (2021). Microtubule-Based Mitochondrial Dynamics as a Valuable Therapeutic Target in Cancer. Cancers.

[5] Reader J.C., Fan C., Ory E.C., Ju J., Lee R., Vitolo M.I., Smith P., Wu S., Ching M.M.N., Asiedu E.B. (2022). Microtentacle Formation in Ovarian Carcinoma. Cancers.

[6] Rajendraprasad G., Eibes S., Boldu C.G., Barisic M. (2021). TH588 and Low-Dose Nocodazole Impair Chromosome Congression by Suppressing Microtubule Turnover within the Mitotic Spindle. Cancers.

[7] Trisciuoglio D., Degrassi F. (2021). The Tubulin Code and Tubulin-Modifying Enzymes in Autophagy and Cancer. Cancers.

[8] Mathias T.J., Ju J.A., Lee R.M., Thompson K.N., Mull M.L., Annis D.A., Chang K.T., Ory E.C., Stemberger M.B., Hotta T. (2022). Tubulin Carboxypeptidase Activity Promotes Focal Gelatin Degradation in Breast Tumor Cells and Induces Apoptosis in Breast Epithelial Cells That Is Overcome by Oncogenic Signaling. Cancers.

[9] Wordeman L., Vicente J.J. (2021). Microtubule Targeting Agents in Disease: Classic Drugs, Novel Roles. Cancers.

[10] Laisne M.C., Michallet S., Lafanechere L. (2021). Characterization of Microtubule Destabilizing Drugs: A Quantitative Cell-Based Assay That Bridges the Gap between Tubulin Based- and Cytotoxicity Assays. Cancers.

[11] Tymon-Rosario J., Adjei N.N., Roque D.M., Santin A.D. (2021). Microtubule-Interfering Drugs: Current and Future Roles in Epithelial Ovarian Cancer Treatment. Cancers.

